# Enhanced recovery after surgery for hip fractures: a systematic review and meta-analysis

**DOI:** 10.1186/s13741-021-00201-8

**Published:** 2021-09-13

**Authors:** Song-yang Liu, Ci Li, Pei-xun Zhang

**Affiliations:** Department of Orthopedics and Trauma, Peking University People’s Hospital, Beijing, China

**Keywords:** ERAS, Hip fracture, Time to surgery, LOS, Complication rate

## Abstract

**Background:**

Enhanced recovery after surgery (ERAS) programs have achieved promising results in many surgical specialties. However, uncertainty still remains regarding the effect of ERAS on hip fractures. The objective of this review was to investigate the clinical prognosis of ERAS programs in terms of (1) hospital-related endpoints (time to surgery [TTS], length of stay [LOS]), (2) readmission rate, (3) complications, and (4) mortality.

**Methods:**

Published literature was searched in the PubMed, EMBASE, and Cochrane Library databases. All of the included studies met the inclusion criteria. The primary outcomes were TTS and LOS. The secondary outcomes included the 30-day readmission rate, overall complication rate, specific complication rate (delirium and urinary tract infection), and 30-day and 1-year mortality. Language was restricted to English. The data analysis was carried out by Review Manager 5.3.

**Results:**

A total of 7 published studies (9869 patients) were finally included, and these were all cohort studies. The meta-analysis showed that the TTS, LOS, and overall complication rate were significantly reduced in the ERAS group compared with the control group (*p* < 0.01). Moreover, no significant change was found in the 30-day readmission rate or 30-day and 1-year mortality.

**Conclusions:**

ERAS significantly decreases the TTS, LOS, and complication rate without increasing readmission rate and mortality, which adds to the evidence that the implementation of ERAS is beneficial to patients undergoing hip fracture repair surgeries.

## Background

As one of the most common injuries in the elderly, hip fracture is predicted to reach 7.3–21.3 million cases around the world by 2050 (Leigheb et al. 2013). Several studies have reported 30-day mortality rates ranging from 6.1 to 8.7% (Bretherton and Parker 2015; Pincus et al. 2017; Sheikh et al. 2017) and 1-year mortality rates ranging from 21 to 30% (Klop et al. 2014; Lund et al. 2014; Mundi et al. 2014). Given the predicted increasing trend of the aging population, hip fractures remain a significant public health concern.

Beside the progress of surgical techniques, some new perioperative care approaches have been developed to improve the outcome and reduce mortality after hip fractures. One example of such an approach is the Enhanced recovery after surgery ERAS program. ERAS was initially advocated in the 1990s by H. Kehlet (1997). ERAS programs address preoperative, intraoperative, and postoperative intervention during the recovery from surgery. The core elements of ERAS include preoperative nutritional support, effective analgesia, optimal pain control, fluid management, postoperative early mobilization and so on (Ljungqvist et al. 2017). The promotion of ERAS has achieved satisfactory results, including a reduction in mortality, length of stay (LOS), and complication rates in many surgical specialties (Bozic et al. 2010; Chen Hu et al. 2012; Larsen et al. 2008). However, its effect and application in hip fracture surgery have not yet been proven.

Hereby, we performed a systematic review and meta-analysis to investigate the clinical prognosis of ERAS programs in terms of (1) hospital-related endpoints (time to surgery [TTS], LOS), (2) readmission rate, (3) complications, and (4) mortality.

## Methods

### Search strategy and criteria

The systematic review of related literature was performed according to Preferred Reporting Items for Systematic Review and Meta-analysis (PRISMA) guidelines (Moher et al. 2009). Two researchers independently searched the PubMed, EMBASE and Cochrane Library databases from January 1966 to July 2020. The language was restricted to English. The search strategy used was as follows: (ERAS or enhanced recovery or fast track) and (hip fractures or femoral fractures or intertrochanteric fractures or subtrochanteric fractures). Reference lists of related papers were also manually searched.

We included studies that implemented ERAS programs in patients undergoing hip fracture surgery. Although the details of ERAS were not unified among these studies, only the studies covering pre-, intra-, and postoperative management of the surgery (Ljungqvist et al. 2017) were included. The exclusion criteria included case reports, editorials, commentaries and reviews.

### Study selection and data extraction

Two authors (Liu and Li) screened the titles and abstracts independently and eliminated duplicates. Both authors reviewed the full texts of the potentially eligible studies and determined the final articles included. Discrepancies were settled by discussion between the two authors. The quality of eligible studies was assessed using the modified Newcastle–Ottawa scale (Stang 2010).

Two review authors extracted the relevant information, including the publication authors, publication years, sample size, age, sex, fracture grade, and follow-up duration. Any inconsistency was either resolved by a third investigator or negotiated between the two original authors.

### Outcome measures

The primary outcomes were time to surgery (TTS) and length of stay (LOS). The secondary outcomes included 30-day readmission rates, 30-day mortality and 1-year mortality, overall complication rate, and specific complication rate (delirium, urinary tract infection, and surgical site infection). Time to surgery was defined as the waiting time from admission to surgery.

### Statistical analysis

**Fig. 1:**
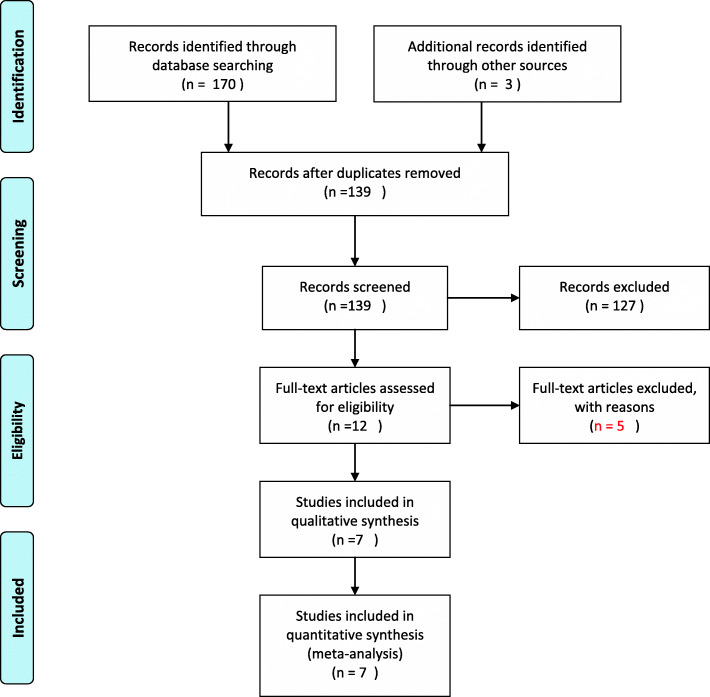
PRISMA flow diagram

Meta-analysis was completed by Review Manager (RevMan, version 5.3). For continuous outcomes (LOS and TTS), the mean difference (MD) with 95% confidence interval (CI) was calculated, while for dichotomous data (the 30-day mortality, readmission, overall and specific complication rate), the odds ratio (OR) was calculated. Statistical heterogeneity was assessed by *I*^2^ measurement as follows: *I*^2^ < 50%: low; 50–75%: moderate; and > 75% high (Higgins et al. 2003). When *I*^2^ < 50%, no significant heterogeneity was indicated, and a random effects model was applied. Otherwise, a fixed effects model was used, and a sensitivity analysis was performed. A *P* value funnel plot was generated to evaluate the publication bias (Fig. 1).

## Results

### Search results and study characteristics

**Fig. 2:**
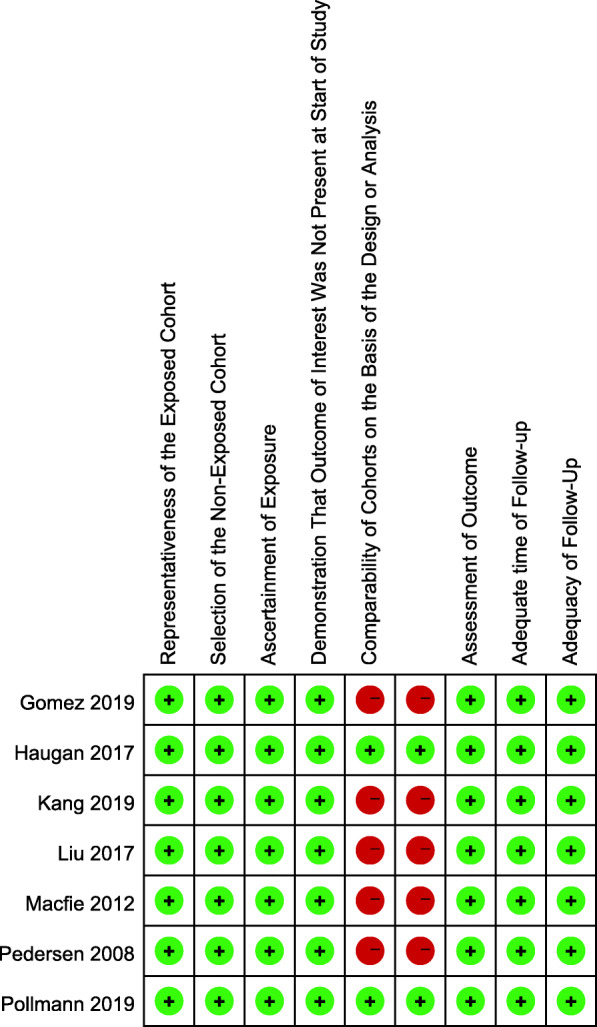
NOS assessment of bias in cohort studies. + low risk of bias, − high risk of bias

The initial literature search generated 173 citations. After removing duplicates, 139 articles underwent title and abstract screening, and 127 were excluded. Following this step, 12 articles were read as full text, and 7 of them (Gomez et al. 2019; Haugan et al. 2017; Kang et al. 2019; Liu et al. 2017; Macfie et al. 2012; Pedersen et al. 2008; Pollmann et al. 2019), involving 9869 participants, met the inclusion criteria and were finally included. All of the included studies were cohort studies. The distributions of age, sex, and types of fracture were similar among the studies. The perioperative fast-track or enhanced recovery processes were all clearly described and were shown in Table 2. The quality of the included studies was evaluated based on the Newcastle-Ottawa Scale (NOS) and was shown in Fig. 2. Only 2 studies in which the confounders were adjusted for during analysis received 9 stars. The remainder achieved 7 stars, as the comparability of cohorts accounted for the majority of bias. The search process is shown in Fig. 1. The basic characteristics of the 7 studies are outlined in Table 1.

**Table 1:** Basic characteristics of the included studies

Studies	Years	Country, Center	Number(E/C)	Mean age(E/C)	Male/Female	Follow-up duration	Type of fracture	Study design
Pollmann (Pollmann et al. 2019)	2019	Single-center, Norway	1140/1090	79.6/79.7	701/1529	1 year	Proximal femur fracture	Cohort study
Kang (Kang et al. 2019)	2019	Single-center, China	50/50	77.81/78.32	31/69	30 d	Intertrochanteric fracture	Cohort study
Gomez (Gomez et al. 2019)	2019	Single-center, France	27/27	84.5/85.0	14/40	1 year	Peritrochanteric fracture	Cohort study
Haugan (Haugan et al. 2017)	2017	Single center, Norway	1032/788	83.1/83.1	512/1308	1 year	Hip fracture	Cohort study
Liu (Liu et al. 2017)	2017	Multicenter, USA	2514/2488	79.7/79.3	1586/3416	30 d	Hip fracture	Cohort study
Macfie (Macfie et al. 2012)	2012	Single-center, Denmark	117/115	82.5/82.7	52/180	6 months	Proximal femoral fractures	Cohort study
Pedersen (Pedersen 2008)	2008	England	178/357	/	127/408	1 year	Hip fracture	Cohort study

### Time to surgery

**Fig. 3:**
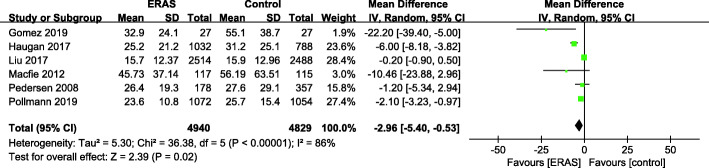
Time to surgery for ERAS versus the control recovery pathway

There were 6 studies (Gomez et al. 2019; Haugan et al. 2017; Liu et al. 2017; Macfie et al. 2012; Pedersen 2008; Pollmann et al. 2019) with available TTS data (Fig. 3). A random effects model was applied, as the heterogeneity was significant (*P* < 0.00001, *I*^2^ = 86%). A significant reduction in the mean TTS was found for the ERAS patients compared with the control group (MD = − 2.96, 95% CI: − 5.40–0.53, *P* = 0.02).

### Length of stay

**Fig. 4:**
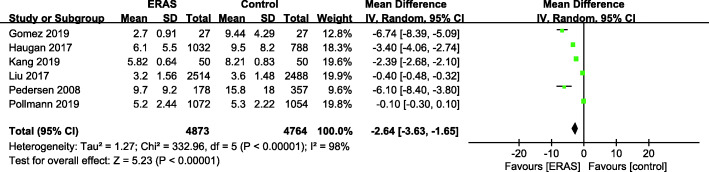
Length of stay for ERAS versus the control recovery pathway

There were 6 studies (Gomez et al. 2019; Haugan et al. 2017; Kang et al. 2019; Liu et al. 2017; Pedersen 2008; Pollmann et al. 2019) with available length of stay (LOS) data (Fig. 4). A random effects model was applied (*P* < 0.00001, *I*^2^ = 90%). A significant reduction in the mean LOS was found for the ERAS patients compared with the control group (MD = − 2.64, 95% CI: − 3.63–1.65, *P* < 0.00001).

### Thirty-day readmission

**Fig. 5:**
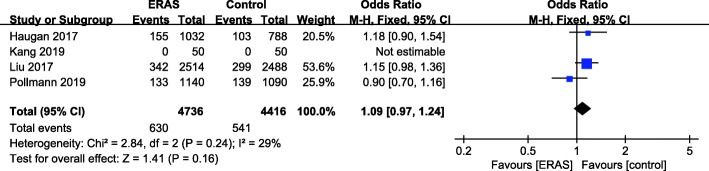
Thirty-day readmission rate for ERAS versus the control recovery pathway

There were 4 studies (Haugan et al. 2017; Kang et al. 2019; Liu et al. 2017; Pollmann et al. 2019) with available overall complication rate data (Fig. 5). A fixed effects model was applied (*P* = 0.24, *I*^2^ = 29%). No increase in the 30-day readmission rate was found for the ERAS group (OR = 1.09, 95% CI: 0.97–1.24, *P* = 0.16) compared with the control group.

### Thirty-day mortality

**Fig. 6:**
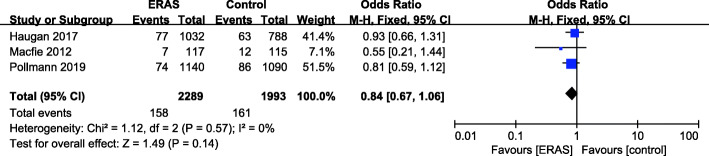
Thirty-day mortality for ERAS versus the control recovery pathway

There were 3 studies (Haugan et al. 2017; Macfie et al. 2012; Pollmann et al. 2019) with available 30-day mortality data (Fig. 6). A fixed effects model was applied (*P* = 0.57, *I*^2^ = 0). No significant reduction in 30-day mortality was found for the ERAS group (OR = 0.84, 95% CI: 0.67–1.06, *P* = 0.14) compared with the control group.

### One-year mortality

**Fig. 7:**
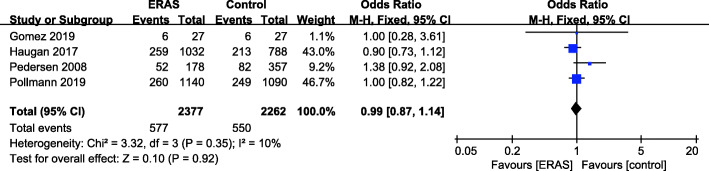
One-year mortality for ERAS versus the control recovery pathway

There were 4 studies (Gomez et al. 2019; Haugan et al. 2017; Pedersen 2008; Pollmann et al. 2019) with available 1-year mortality data (Fig. 7). A fixed effects model was applied (*P* = 0.35, *I*^2^ = 10%). No significant reduction in 1-year mortality was found for the ERAS group (OR = 0.99, 95% CI: 0.87–1.14, *P* = 0.92) compared with the control group.

### Overall complication rate

**Fig. 8:**
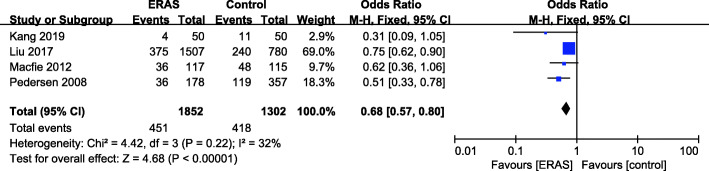
Overall complication rate for ERAS versus the control recovery pathway

There were 4 studies (Kang et al. 2019; Liu et al. 2017; Macfie et al. 2012; Pedersen 2008) with available overall complication rate data (Fig. 8). A fixed effects model was applied (*P* = 0.22, *I*^2^ = 32%). A significant reduction in the overall complication rate was found for the ERAS group (MD = 0.68, 95% CI: 0.57–0.80, *P* < 0.00001) compared with the control group.

### Delirium rate

**Fig. 9:**
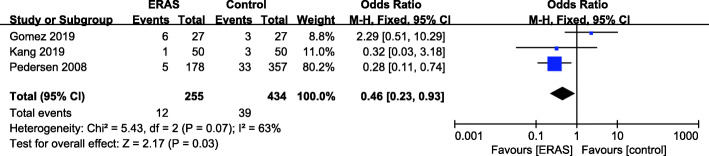
Delirium rate for ERAS versus the control recovery pathway

There were 3 studies (Gomez et al. 2019; Kang et al. 2019; Pedersen 2008) with available delirium rate data (Fig. 9). A random effects model was applied (*P* = 0.07, *I*^2^ = 63%). A significant reduction in the delirium rate was found for the ERAS group (OR = 0.46, 95% CI: 0.23–0.93, *P* < 0.03) compared with the control group.

### Urinary tract infection rate

**Fig. 10:**
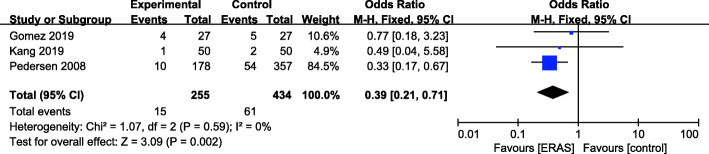
Urinary tract infection (UTI) rate for ERAS versus the control recovery pathway

There were 3 studies (Gomez et al. 2019; Kang et al. 2019; Pedersen 2008) with available urinary tract infection (UTI) rate data (Fig. 10). A fixed effects model was applied (*P* = 0.59, *I*^2^ = 0). A significant reduction was found for the ERAS group (OR = 0.39, 95% CI: 0.21–0.71, *P* = 0.002) compared with the control group.

### Publication bias

**Fig. 11:**
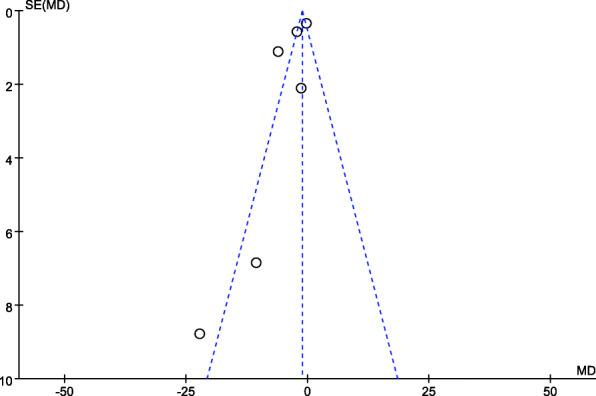
Funnel plot using TTS as an indicator

A funnel plot was used with TTS as an indicator. The 6 studies were distributed asymmetrically in the plot, which suggested a high impact of publication bias on the results (Fig. 11).

## Discussion

This is the first meta-analysis to investigate the effects of the ERAS protocol among hip fracture patients. The primary outcome measures, including LOS and TTS, were significantly reduced in patients treated with the ERAS program. Of equal importance, the overall complication rate, delirium rate, and UTI rate were also significantly reduced, while the 30-day readmission rate and mortality (30 days and 1 year) were not increased compared to those of the control groups. Our results were consistent with previous meta-analytical findings in other surgical specialties, such as hepatectomy (Ni et al. 2015), colorectal surgery (Greco et al. 2014), and pancreatic surgery (Coolsen et al. 2013).

In Table 2, we summarize the various ERAS protocols performed in each study. Despite the diversity of ERAS protocols during pre-, intra-, and postoperative stages, some core elements were shared in common. The most commonly emphasized elements include nutrition therapy (protein drinks), opiate-sparing pain relief, avoidance of drains, oxygen therapy, and early mobilization. These details might serve as a reference for future guidelines of standard ERAS programs in hip fracture surgery.

**Table 2:** ERAS elements in each article

Article	Preoperative	Intraoperative (and surgery)	Postoperative
Pollmann (Pollmann et al. 2019)	Intravenous fluids Oxygen Opiate-sparing pain relief Electrocardiogram Triage of hip fractures X-ray evaluation Fascia iliaca compartment block Short periods of fasting	Transfusion triggers Management of anticoagulants	Pain relief Standardized mobilization Screening for nutritional status Prevention of delirium
Kang (Kang et al. 2019)	Preoperative educational program Oral multimodal analgesia (Celebrex) Opioid-free spinal anesthesia	Intravenous dexamethasone 2 L of lactated Ringer’s Tranexamic acid	Early mobilization Opioids avoiding Nausea and vomiting control Supported discharge
Gomez (Gomez et al. 2019)	Geriatrician support Nursing aids Physical therapy	General anesthesia with an ultrasound-guided femoral nerve block No drains Reduction on traction table	Early food supply Transfer to postacute rehabilitation (PAR)
Haugan et al. (Haugan et al. 2017)	Oxygen Standardized nursing routines (pain control, nutrition, fluid therapy and prevention of pressure sores) Femoral block Preparation for discharge Scheduled time for surgery (within 24 hours)	Not mentioned	Standardized pain control Standardized mobilization Medication reconciliation
Liu (Liu et al. 2017)	Patient education No prolonged fasting Carbohydrate loading Decreased sedative medications Regional anesthesia	Antimicrobial prophylaxis nausea and vomiting prophylaxis Multimodal analgesia Standard anesthetic protocol (neuraxial anesthesia preferred) Minimally invasive surgery Avoidance of drains and tubes Perioperative fluid management Prevention of hypothermia	Multimodal analgesia Early oral nutrition Early and sustained ambulation Early urinary catheter removal Deep vein thrombosis prevention Restoration of gut function Chewing gum (colorectal)
Macfie et al. (Macfie et al. 2012)	Patient information sheet Early preassessment Fascia iliaca block and optimal analgesia Limited fasting and carbohydrate loading	High inspired oxygen Optimal fluid management Avoidance of drain	Postoperative early mobility Perioperative nutritional support Breathing exercise
Pedersen et al. 2008	Specialized hip fracture ward Pain treatment Femoral nerve block Assessment by anesthesiologist Planning of fluid therapy Blood sampling Immediately X-rays evaluation Oxygen therapy	Spinal anesthesia	Specialized hip fracture ward Oxygen therapy Nutrition therapy (protein drinks) Early mobilization

### Time to surgery

Current guidelines suggest that surgery be completed within 48 h of hip fracture (Brox et al. 2015), as many clinical trials reported that a longer time to surgery was associated with a longer hospital stay (Al-Ani et al. 2008), higher mortality (Nyholm et al. 2015), increased risk of infection (Westberg et al. 2013), and other complications (Petersen et al. 2006). A high-quality meta-analysis also showed that earlier surgery and shortened time to surgery after hip fracture were associated with a lower risk of death and lower rates of postoperative complications among hip fracture patients (Simunovic et al. 2010). In our review, we found that the mean time to surgery was reduced by 2.96 h in ERAS patients compared with the control group, which might exert a positive effect on the postoperative and complication rates.

### Length of stay

The average LOS after hip fracture ranges widely from 5.6 to 45 days among different countries (Ireland et al. 2015; Nikkel et al. 2015; Nordstrom et al. 2015; Sund et al. 2011). It was reported that prolonged LOS was related to an increased rate of healthcare-associated infections and some postoperative complications, such as delirium (Mosk et al. 2017). In addition, Nikkel et al. found that decreased LOS was related to reduced rates of early mortality (Nikkel et al. 2015). Furthermore, reduction of LOS is a reliable way to save tremendous amounts of hospital resources and costs (Kaoutzanis et al. 2018). Thus, numerous methods and ongoing efforts have been made to reduce the LOS.

ERAS has been demonstrated to be an effective way of reducing LOS. The current review showed that the mean reduction in LOS in ERAS patients was 2.64 days (95% CI:) compared to that in controls, which was not only statistically significant but also clinically meaningful. The data are consistent with the findings in several other surgical specialties. Zhu et al. conducted a meta-analysis to evaluate the effects of ERAS on hip and knee arthroplasty and found that LOS was significantly lower in the ERAS group than in the control group (SMD = − 0.85, 95% CI: − 1.24 to − 0.45, *P* = 0.01) without an increase in the 30-day readmission rate (Zhu et al. 2017). The results of a meta-analysis of noncolorectal abdominal surgery indicated a significant reduction (2.5 days) in the mean LOS for ERAS patients compared with the control group (Visioni et al. 2018). In addition, the reduction of time to surgery, optimized nutrition and fluid management, sufficient pain control, and early mobilization in the ERAS protocol comprehensively explain the reduced LOS in our review.

### Thirty-day readmission rate

Thirty-day readmission is another important indicator used to evaluate the quality of surgery. Early readmission after hip fracture is associated with increased mortality and worse postoperative outcomes (French et al. 2008; Kates et al. 2015a, 2015b). Furthermore, a high readmission rate imposes a heavy financial burden on the healthcare system (Jencks et al. 2009; Kates et al. 2015a, 2015b). Therefore, growing attention has been drawn to investigating the risk of readmission and reducing the 30-day readmission rate after hip fracture surgery.

As mobilization and rehabilitation after discharge are of equal importance to hip fracture patients, some researchers are concerned that a shortened LOS is associated with an increased 30-day readmission rate (Capelastegui et al. 2008). The overall readmission rate in our review was 13.3%, which is similar to the readmission rate (10%) of a large-scale trial including 8434 hip fracture patients (Basques et al. 2015). It should be pointed out that the reduction in LOS was not at the expense of an increased readmission rate in our review (OR = 1.09, 95% CI: 0.97–1.24, *P* = 0.16). This result was consistent with several previous articles. A meta-analysis by Zhu et al. showed no significant difference in the 30-day readmission rate (*P* = 0.18) between ERAS and control groups among patients undergoing low extremity arthroplasty (Zhu et al. 2017). Visioni et al. (2018) reported no significant increase in the readmission rate in noncolorectal abdominal surgery. Khan MA reported that the most common causes of readmission after hip fracture were pneumonia, dehydration and renal dysfunction, and deteriorating mobility (Khan et al. 2012). More precise ERAS protocols to minimize these pre- and postoperative risk factors need to be panned and implemented.

### Overall complication rate

The pain, bleeding, immobility, active inflammation, hypercoagulable status, and stress states resulting from hip fractures always precipitate various complications (Beloosesky et al. 2007; Chuang et al. 2005; Desborough 2000). The common complications of hip fractures include delirium, urinary tract infection (UTI), pneumonia, VTE, and surgical site infection (Investigators 2020).

Several studies have demonstrated that ERAS can decrease the overall complication rate (Varadhan et al. 2010; Zhu et al. 2017). Consistent with their findings, our findings revealed a significant reduction in the overall complication rate in the ERAS group (OR = 0.68, 95% CI: 0.57–0.80, *P* < 0.00001) compared with the control group. Due to the lack of data, we only chose delirium and UTI for specific complication analysis. The results show that the UTI and delirium were significantly reduced in our review. A recent large-scale randomized controlled trial (RCT) (Investigators 2020) indicated that surgery within 6 h significantly reduced delirium and UTI, which is closely consistent with our findings. Considering all these results, we could conclude that the shortened time to surgery might reduce the risk of complication rate by reducing urinary tract infection, controlling pain, and getting these patients mobilized earlier than patients assigned to control groups.

### Mortality

Interestingly, despite the shortened time to surgery, reduced LOS, and decreased overall complication rate, no significant change was found in either 30-day mortality or 1-year mortality. It has been reported that the 1-year mortality could reach 30% after hip fracture (Lund et al. 2014). Numerous factors contribute to mortality after hip fracture. A meta-analysis conducted by Chang et al. identified the time to surgery, residential status, cardiovascular disease, pulmonary disease, and malignancy as preventable risk factors significantly associated with mortality (Chang et al. 2018). Toby Smith et al. reported that the four key characteristics associated with the risk of 1-year mortality were abnormal ECG, cognitive impairment, age > 85 years, and mobility before surgery (Smith et al. 2014). In summary, both the time to surgery and the preoperative characteristics of the patients could affect mortality after hip fracture. This partly explains why in our review, the time to surgery was decreased but the mortality remained unchanged between the ERAS and control groups, as most included studies did not adjust the patients’ preoperative status for analysis. In addition, the reduction of 2.96 h might not reach the cutoff that could exert an effect on mortality. However, an observational study including 4500 patients who underwent hip and knee replacement showed that 2-year mortality was significantly reduced after the introduction of ERAS (Savaridas et al. 2013). The long-term effect of ERAS on hip fracture needs to be further investigated.

### Heterogeneity

The heterogeneity in the outcome of “length of stay” and “time to surgery” was high (*I*^2^ > 75%). We conducted a sensitivity analysis to explore the reason for the high heterogeneity. One study was removed at a time to evaluate the influence of the deleted study on the overall result. However, the heterogeneity remained high regardless of which study was removed. The lack of uniform guidelines leads to diverse means of intervention during the implementation of the ERAS protocol, which might contribute to the high heterogeneity. In addition, the funnel plot was asymmetric, which suggested that there might be publication bias (Fig. 11). Such publication bias might be another reason for the high heterogeneity.

### Limitation

There are several limitations in our review. First, the heterogeneity of some final outcomes was relatively high, as considerable variations in surgical techniques and preoperative patient comorbidities existed among the included studies. Furthermore, the high heterogeneity across the available trials hindered definitive statements, and additional, precisely designed studies are required in this area. Last, no high-quality RCTs met the inclusion criteria, which might cause risks of bias. Thus, the findings in our review should be interpreted cautiously with inherent limitations.

## Conclusion

The results of the meta-analysis showed that the implementation of ERAS can significantly decrease the time to surgery, LOS, and overall complication rate without increasing the 30-day readmission rate and mortality in hip fracture patients. These findings add the value of ERAS to the evidence-based database and indicates that the implementation of a standard ERAS protocol is greatly beneficial to patients with hip fracture. In addition, our results also indicate that future trials should focus on standardized ERAS implementation and its long-term effects on improving the quality and value of surgical care in cases of hip fracture.

## Data Availability

All data generated or analyzed during this study are included in this published article and its supplementary information files.

## Abbreviations

CI: Confidence interval
ERAS: Enhanced recovery after surgery
LOS: Length of stay
MD: Mean difference
OR: Odds risks
PRISMA: Preferred Reporting Items for Systematic Reviews and Meta Analyses
RCT: Randomized controlled trial
TTS: Time to surgery
UTI: Urinary tract infection
